# Electroencephalogram abnormalities in panic disorder patients: a study of symptom characteristics and pathology

**DOI:** 10.1186/1751-0759-4-9

**Published:** 2010-08-23

**Authors:** Karin Hayashi, Mariko Makino, Masahiro Hashizume, Koichi Nakano, Koji Tsuboi

**Affiliations:** 1 Department of Neuropsychiatry, Sakura Medical Center, Faculty of Medicine, Toho University, Chiba, Japan; 2 Department of Psychosomatic Medicine, Faculty of Medicine, Toho University, Tokyo, Japan; 3 Support Center for Research and Education, Toho University, Tokyo, Japan

## Abstract

**Background:**

Since the 1980s, a high EEG abnormality rate has been reported for patients with panic disorder. However, how the EEG abnormalities of panic disorder patients are related to the clinical features and pathology of these patients has yet to be clarified. In this study we investigated whether or not EEG abnormalities are related to the 13 symptoms in the DSM-IV criteria for a diagnosis of panic attacks.

**Methods:**

Subjects were 70 patients diagnosed with panic disorder.

Logistic regression analysis was performed with EEG findings as dependent variables and age, sex and with or without the 13 symptoms as independent variables.

**Results:**

(1)EEG findings for panic disorder patients with EEG abnormalities: Of the 17 patients, 13 had repeated slow waves in the θ-band; the most prevalent EEG abnormality found in this study. Paroxysmal abnormality interpreted as epileptiform was found in only two cases. (2)Nausea or abdominal distress (37.7% vs 82.45%, OR-12.5), derealization or depersonalization (7.5% vs 47.1%, OR = 13.9,) and paresthesias (43.4% vs 64.7%, OR = 7.9,) were extracted by multivariate analysis as factors related to EEG abnormalities.

**Conclusion:**

Of the 70 patients studied, 17 had EEG abnormalities. Among these 17 cases, "repeated slow waves in the θ-band" was the most common abnormality. The factors identified as being related to EEG abnormalities are nausea or abdominal distress, derealization or depersonalization, and paresthesias. The study indicated that physiological predispositions are closely related to panic attacks.

## Background

Recently, panic disorder causing panic attacks characterized by symptoms including unexpected palpitation, dyspnea, dizziness, and paresthesias has increasingly been consulted in various kinds of clinics and hospitals. * Katon and colleagues *reconfirmed that 6.7% of primary care patients meet the diagnostic criteria for panic disorder (PD) [1]. It is also reported that 28% of PD patients consult emergency rooms (ER) [2]. The lifetime prevalence of PD is 0.4% in Taiwan [3]. According to National Comorbidity Survey (NCS) data, the life prevalence of PD in the United States is 3.5% while the life prevalence of panic attack (PA) is 7.3% [4]. In Japan, *Kaiya *reported that out of 4,000 subjects investigated, the prevalence among subjects whose response met the criteria for PA was 6.6% while the prevalence among those whose response met the criteria for PD was 5.0%[5].

The initial manifestation age of panic disorder is in one's twenties and the risk in women is twice as high as in men [6]. In panic disorder, anticipatory anxiety and agoraphobia continue even between panic attacks and patients can be operationally diagnosed as having panic disorder from clinical symptoms. However, PD has another aspect that has been understood as a brain problem rather than a mental problem because it is induced when the sodium lactate level increases. Recent studies on PD in brain science suggest that some pathologic brain conditions such as lesions in the amygdala and hippocampus are deeply related to the disorder and other reports hold the coincident view that blood flow decrease in the prefrontal cortex is involved [7-10].

Furthermore, according to genetic studies, the hereditability of panic disorder is in the range of 35-40% with concordance in identical twins being higher than that in fraternal twins. However, many studies report concordance in identical twins to be 50% or less. The above suggest that both genetic disposition and environmental factors are related to panic disorder [11-13].

Since the 1980s, a high EEG abnormality (15-30%) has been reported for patients with PD [14-17]. We carried out this study to investigate how the EEG abnormalities of PD patients are related to the clinical features and pathology of these patients. The risk of diagnosing panic disorder as epilepsy has been pointed out by some specialists. There are a few case studies in which patients who had been initially diagnosed with panic disorder later proved to have been suffering from epilepsy[18,19]. This misdiagnosis risk could be attributed to the fact that of the 13 symptoms in the diagnosis criteria of panic attack in the Diagnostic and Statistical Manual of Mental Disorders(DSM)-IV, 12 symptoms are also observed in partial epilepsy[20,21]. Feeling of chocking is the only exception. It should be noted that epilepsy is diagnosed operationally, while an EEG check is only supplementary.

We have studied PD which shows EEG abnormalities, and found that some of the EEG abnormalities were related to PD. Some other subjects of our study had a significantly higher rate of "hypersensitivity to strong lights or flashes" and "conversions anamnesis" than patients without EEG abnormalities.

The objective of this study is to investigate whether or not EEG abnormalities are related to the 13 symptoms in the DSM-IV criteria for a diagnosis of panic attacks and to consider how panic disorder and EEG abnormalities relate to each other.

## Methods

### [Subjects]

The Subjects consisted of 903 males (age 40.7 ± 16.6) and 1,416 females (age 41.1 ± 18.5) who visited for the first time the Department of Psychosomatics Medicine at Toho University Omori Medical Center in the period from February 2007 to July 2008. First, subjects were examined by interview doctors to confirm that they had the 13 symptoms used in the diagnosis criteria of panic attack in DSM-IV. Second, psychosomatic medicine specialists excluded physical diseases such as arrhythmia, angina, hyperthyroidism, chronic obstructive pulmonary disease (COPD), asthma, pheochromocytoma, and neurological disorders, including evident epilepsy. Subjects were informed of the medical significance [22,23] of EEG for all patients diagnosed with panic disorder. Finally, 115 subjects between the ages of 18-65 who gave consent to EEG check were selected. The list was further shortened to 70 subjects (20 males, age 33.2 ± 8.2 and 50 females, age 35.0 ± 9.5) when the following groups were excluded:

1) Patients who regularly take psychotropic drugs and other medicines with EEG effects 2) Patients with schizophrenia, severe depression, and personality disorders

3) Patients with alcoholism or drug abuse

4) Patients with severe complications of circulatory, respiratory, digestive, endocrine and neurological disease.

The selected 70 subjects were made aware of the purpose and methods of the study. They were assured that study data would be anonymously and statistically treated. It was also made clear that personal data would not be disclosed to anyone including administrative authority. Questions from subjects were readily answered and subjects were given the choice to decline to participate in the study without fearing any consequences.

The Ethics Committee of Faculty of Medicine Toho University approved this study. They also approved EEG check of panic disorder patients within normal medical examination procedures. Furthermore, to avoid delay in the commencement of treatment, EEG technicians were advised to check patients EEG on the first examination day.

### [EEG record]

For EEG record, Nihon Kohden EEG-1514 leads were attached to both earlobes (A1 and A2). The reference electrode was 12 channels: Fp1, Fp2, C3, C4, P3, P4, 01, 02, F7, F8, T3 and T4, according to the international 10-20 system of Electrode Placement, and the EEG was recorded for 15 consecutive minutes or more. The bipolar leads were 12 channels: Fp1-F3, Fp2-F4, F3-C3, F4-C4, C3-P3, C4-P4, P3-01, P4-02, Fp1-F7, Fp2-F8, F7-T3 and F8-T4, and the EEG was recorded for 2 consecutive minutes or more. Photic stimulation was given at 10 second intervals at 3, 5, 6, 8, 10, 12, 14, 15, 18, 20 and 24 Hz, and hyperventilation was carried out for 5 minutes at 3 second intervals. The time constant was 0.3 seconds by high cut filter 120 Hz and the contact resistance was 10 kΩ or less.

### [EEG record reading and interpretation]

EEG record reading was based on the decision criteria of adult EEG proposed by *Teruo Ohkuma *in 1999, which are widely accepted in Japan. The criteria details are 1) EEG with eyes closed is composed of α wave or faster activity than α wave. Obvious θ and δ waves do not appear. 2) α waves and fast activity show normal localization. 3) There is no difference of 20-30% or more in the amplitude of symmetric parts. 4) There is no difference of 10% or more in the duration of symmetric parts. 5) α attenuation occurs with open eyes, sensory stimuli, and mental activities et cetera. 6) Neither α wave nor fast activity show abnormally high amplitude. 7) No intermittent activities appear, such as spike waves or sharp waves(intermittent abnormal activity, epileptic pattern.

We used the criteria and the author read all EEG records of patients before contact with them. The peculiar EEG patterns which are difficult to read or with clinical significance unknown at present (not taken as abnormal findings in reading EEG), such as 14 & 6 Hz positive spike, small sharp spikes, 6 Hz spike and slow waves, psychomotor variant, SREDA (subclinical rhythmic electroencephalographic discharge of adults) and Wicket spikes were considered normal as long as they were not frequent and their basic activity's localization, rhythmicity and consecutiveness were stable [24,25]. The interpretation of the EEGs was finalized after double-checking by my executive doctor.

### [Statistical analyses]

Logistic regression analysis was performed with EEG findings as dependent variables and with or without the 13 symptoms above, age and sex were independent variables.

We confirmed there was no significant difference with agoraphobia, psychiatric disorders, drinker, and smoker or not (Table 1). The independent variables were selected stepwise and by forward selection under the likelihood ratio testing was selected. The SPSS version 13 was used for statistical analysis.

**Table 1:** Characteristics of patients with panic disorder subclassified on the basis of EEG findings

	EEG normal(M/F)	EEG abnormal(M/F)
Panic Disorder	53(17/36)	17(3/14)
With agoraphobia	33(11/22)	14(3/11)
With other recent psychiatric disorders		
Depression	6(3/3)	2(0/2)
Somatoform disorder	2(0/2)	1(0/1)
With other past psychiatric disorders		
Depression	7(2/5)	4(1/3)
Somatoform disorder	0	0
Smoker	22(8/14)	3(0/3)
Drinker	9(5/4)	2(2/0)

## Results

(1) EEG findings of panic disorder patients with EEG abnormalities (see Table 2)

**Table 2:** EEG abnormal findings of 17 cases

**No**.	age	sex	Basic activity	Abnormal findings	Focus	Build up
1	26	female	9-10 Hz irregular	Slow wave burst (θ δ)	PO~diffuse	+

2	59	female	10-12 Hz irregular	Slow wave burst (θ)	CPO	Cannot check

3	40	female	10 Hz regular	Slow wave burst (θ)	Right CPO	-

4	26	female	9 Hz regular	Slow wave burst (θ)	Diffuse	-

5	18	female	10-11 Hz regular	Sharp & slow wave complex *1	Right	-

6	33	female	10-11 Hz regular	Slow wave burst (θ)	CPO	-

7	36	female	10-11 Hz regular	Slow wave burst (θ)	CPO	-

8	35	female	9 Hz irregular	Slow wave burst (θ)	CPO	-

9	37	female	10-12 Hz regular	Slow wave burst (θ)	Diffuse	-

10	23	female	9-11 Hz regular	Sharp wave (6 hz)	CPO	+

11	31	male	12 Hz regular	Slow wave burst *2	PC	+

12	35	male	8-10 Hz irregular	Slow wave burst (θ) Positive spike waves	Diffuse Right Fp	+

13	34	female	8-10 Hz irregular	Slow waves (7 hz)	intermingled at basic activity	-

14	38	female	10-11 Hz regular	Positive sharp waves	Left CPO	+

15	39	male	10 Hz regular	Slow waves (6-7 hz)	intermingled at basic activity	+

16	47	female	10-11 Hz regular	spike & slow wave complex	Right	+

17	19	female	10-12 Hz irregular	Slow wave bursts (6-7 hz)	Diffuse	+

*1) the EEG waves of this case were shown in Figure 2

**Figure 1:**
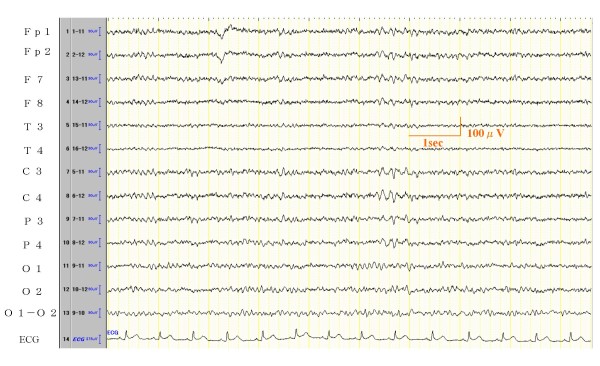
**EEG record of Case 11**. EEG Result: Basic activity is 12~13 Hz 20~50 μV with regular α waves. 6-7 Hz θ wave activities appear in the posterior and central channels occasionally. Judgment of this EEG is slightly abnormal.

*2) the EEG waves of this case were shown in Figure 1

The most prevalent EEG abnormalities were slow waves bursts temporally repeated, found in 11 of 17 cases (representative EEG waves in Figures 1). There was no localization. Some of them had no difference between right and left, however 4 cases were dominant in the right side.

**Figure 2:**
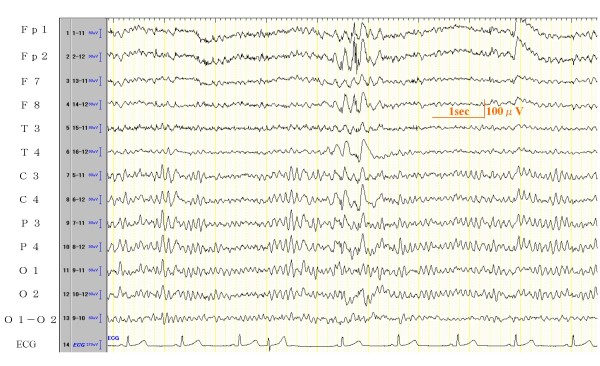
**EEG record of Case 5**. EEG Result: Basic activity is 10-11 Hz 30~100 μV with regular or irregular α waves. Sharp & slow wave complexes appear in the right a few times. Judgment of this EEG is slightly abnormal.

Paroxysmal abnormality interpreted as epileptiform was found only in 2 cases: No. 5 (Figure 2) and No. 16. The positive spike waves in No. 12 and the positive sharp waves in No. 14 were interpreted as abnormal because they had a difference between right and left side and were repeating in the same region. No slow wave complex was found and amplitudes were low or medium, therefore they were not considered as epileptiform discharges. In 8 of the 17 cases, an evident build up was found by hyperventilation, although all patients recovered in 30 seconds.

(2) The 13 symptoms in the DSM-IV diagnosis criteria and EEG findings (see Table 3)

**Table 3:** Comparison of 13 symptoms with the EEG findings of panic disorder patients

	EEG normal patients = 53(%)	EEG abnormal patients = 17(%)
palpitations, pounding heart, or accelerated heart rate	44 (83.0)	13 (76.5)
sweating	11 (20.8)	5 (29.4)
trembling or shaking	16 (30.2)	5 (29.4)
sensations of shortness of breath or smothering	40 (75.5)	13 (76.5)
feeling of choking	13 (24.5)	4 (23.5)
chest pain or discomfort	22 (41.5)	7 (41.2)
**nausea or abdominal distress**	**20 (37.7)**	**14 (82.4)**
feeling dizzy, unsteady, lightheaded, or faint	38 (71.7)	15 (88.2)
**derealization or depersonalization**	**4 (7.5)**	**8 (47.1)**
fear of losing control or going crazy	23 (43.4)	11 (64.7)
fear of dying	16 (30.2)	5 (29.4)
**paresthesias**	**23 (43.4)**	**11 (64.7)**
chills or hot flushes	11 (20.8)	7 (41.2)

age	34.6 ± 9.3	33.9 ± 9.8
sex	M:17 F:36	M: 3 F:14

Nausea or abdominal distress (37.7% vs 82.45%, OR = 12.489, 95%CI: 2.422-64.426, p = 0.003); derealization (feelings of unreality) or depersonalization (being detached from oneself) (7.5% vs 47.1%, OR = 13.919, 95%CI: 2.579-75.083, p = 0.002); or paresthesias (numbness or tingling sensations) (43.4% vs 64.7%, OR = 7.928, 95%CI: 1.567-40.082, p = 0.012) were extracted by multivariate analysis as factors related to EEG abnormalities (Table 4).

**Table 4:** EEG findings of panic disorder patients by the three factors extracted by multiple regression analysis.

	EEG normal patients = 53(%)	EEG abnormal patients = 17(%)	Wald	OR	95%CI	p
nausea or abdominal distress	20 (37.7)	14 (82.4)	9.102	12.489	2.422-64.426	0.003
derealization or depersonalization	4 (7.5)	8 (47.1)	9.369	13.919	2.579-75.083	0.002
paresthesias	23 (43.4)	11 (64.7)	6.261	7.928	1.567-40.082	0.012

## Discussions

### [EEG abnormality rate in PD patients]

In the 1990s, it was often said that many panic disorder patients had abnormal EEG[15-17]. On the other hand, *Stein et al *could not find many EEG abnormalities in their patients with PD (14.3%). This was a descriptive epidemiological study. In their study, however, the number of cases was 35, which is considered small. Moreover, all EEGs were read by the neurologists who were directly examining the patients, which may have caused their decision criteria to be varied or their conclusions to be biased [14].

The study of *Lepola et al*. was part of a larger trial studying "the effect of psychotropic medication on panic disorder". It was a descriptive epidemiological study in which 54 consecutive inpatients were included. The aim of the study was to investigate the EEGs of a large number of panic patients in non-medicated condition and to compare the EEG with Computed Tomography (CT) results. In this study 13 patients, 24%, displayed abnormal EEG recordings, and 6 patients, 20%, exhibited abnormal CT scans [15].

The study of *Dantendorfe et al*. invited public participation of 120 subjects gathered through general practitioners and media. It was a case-control study in which 35 patients, 29.2% of the patients examined, showed EEG abnormalities. In this study, the panic disorder diagnosis was carried out by two independent psychiatrists and 40% of the subjects were under medication. EEGs were read by only one psychiatrist who had no relationship with the subjects [16].

The study of *Bystrisky et al*. was a part of the "Clinical trial examining the efficacy of clonazepam in the treatment of panic disorder" where patients use of psychotropic medication was strictly confirmed by blood and urine toxicology screens. 21 panic disorder patients together with 20 healthy volunteers participated in this case-control study. In this study, 5 patients, 23.8%, showed EEG abnormalities. Patients with panic disorder in this study tended to have less alpha power in the right temporal region [17]. However, the EEGs were read by only one psychiatrist, without showing the decision criteria, and there were no double-checks.

Of the 70 cases with panic disorder who participated in this study, 17 patients, 24.3%, were designated as abnormal in the final interpretation of EEG. The EEG abnormality rate reported for healthy people is in the range of 4.9-10%[26-28]. Our findings also showed a high EEG abnormality rate among patients with panic disorder, although we had no control subjects.

### [Panic disorder and epilepsy]

When considering the EEG abnormalities found in many panic disorder patients, epilepsy, the symptoms of which are similar to those of panic disorder, has to be carefully considered. In fact, comorbidity between panic disorder and epilepsy has been pointed out [23]. Recent studies associate epilepsy with an increased prevalence of anxiety disorder compared with the general population [29-31]. According to *Tellez-Zenten*, the lifetime prevalence of panic disorder in people with epilepsy was 6.6%. In people without epilepsy it was 3.6%[30]. *Akanuma *reported that 26.7% of idiopathic generalized epilepsy (IGE) patients were comorbid anxiety-panic disorder[31]. However we couldn't find the epidemiological evidence data of the prevalence of epilepsy in panic disorder populations. There are many reports about cases which had been initially diagnosed as panic disorder and later diagnosed as epilepsy[18,19], and the conclusion is that patients with panic disorder or epilepsy need to be examined in a careful way. There were some cases of repeated EEG and morphological tests (such as magnetic resonance imaging (MRI), CT, etc.), however we cannot tell panic disorder from epilepsy. For these patients, it is beneficial to consider EEG abnormalities in consecutive treatments [32]. According to *Masnou*, prescription of anticonvulsants is good, however, side effects and the condition of the patients needs to be observed carefully [19]. It should be mentioned that there were cases where panic disorder patients had been misdiagnosed with epilepsy and anticonvulsants had been prescribed for years[33,34].

There could be a risk that iatrogenic side effects may be caused in panic disorder and epilepsy. However, most of this literature is just case reports. Many of the actual EEG abnormality findings do not originate from epilepsy, although the EEG abnormality rate is high in panic disorder patients. Therefore, "epilepsy" is considered an important differential factor in the diagnosis of panic disorder; however, EEG abnormality cases are not always epilepsy.

### [EEG abnormal findings in panic disorder]

All abnormal EEG findings were non-specific slow waves in *Lepola *and *Dantendorfer's *studies [15,16]. In the study of Stein, three of five were non-specific slow waves and two were paroxysmal abnormalities that could not be identified clearly as epileptiform discharges [14].

In the study of *Bystritsk*, there were 25% EEG abnormal patients, and 15% of the patients had slow wave activity in the temporal regions with occasional bursts of sharp waves identified as epileptifom discharges. In addition, 10% of them had nonspecific increases in generalized slow wave activity [17]. Thus there is no consensus regarding EEG abnormality findings in panic disorder patients.

In our study, 13 cases had slow wave abnormalities which intermingled in intermittent slow activity and continuous slow activity, and only 2 of the 17 cases had paroxysmal abnormalities that were interpreted as epileptiform discharges. *Daly *and *Bagchi et al*. found in their EEG check that paroxysmal slow activity (similar slow wave burst) which were poor in locality reflected abnormalities in the brainstem or the deep brain near the brainstem, such as the thalamus, mesencephalon, medial frontal lobe, posterior cranial fossa, and thalamocele [35,36]. However, paroxysmal slow activities are sometimes found in widespread lesions over both the cerebral cortex and subcortical grey matter [37]. Also, in 1981 paroxysmal slow activity was considered to be a non-specific abnormality because it was difficult to infer the abnormal region [38].

### [EEG abnormalities in panic disorder]

Based on the findings, it was considered that

i. Many panic disorder patients had EEG abnormalities.

ii. It is important to make a differential diagnosis between panic disorder and epilepsy since they are intricately interrelated to each other and have clinical similarities. In this study, however, only two out of the 70 cases examined had epileptiform discharges. Accordingly, we could not confirm the rate of epilepsy cases to be high.

iii. The high rate of EEG abnormalities in panic disorder patients might have some relationship to physiological predispositions that easily cause panic attacks.

### [EEG abnormalities in panic disorder and the 13 symptoms]

This is the first study that analyzed panic attack symptoms by dividing them into EEG normal and abnormal groups. In this study, 3 of the 13 symptoms, nausea or abdominal distress, derealization(feelings of unreality) or depersonalization (being detached from oneself), and paresthesias (numbness or tingling sensations) were extracted by multivariate analysis as factors related to EEG abnormalities in panic disorder.

In the past, *Stein et al*. could not find any relation between EEC abnormalities and psychosensory symptoms such as derealization, depersonalization, visual or auditory perceptual disturbances and forced thinking [14]. *Weilburg et al*. reported that they carried out EEG monitoring in 15 patients and found EEG changes during panic attacks, but could not extract any specific symptoms which might be related to EEG abnormalities [22]. Both *Stein et al *and *Weilburg et al *expected the existence of clinical symptoms related to EEG abnormalities and tried to investigate them in vain. Their efforts were not successful because number of subjects in their studies was small - 35 and 15 respectively -. In our study the number of subjects was 70, and the following three symptoms were extracted as factors related to EEG abnormalities.

1. Nausea or abdominal distress

One of the mechanisms of this symptom is the direct stimulations of the vomiting center in the medulla. EEG abnormality might be a factor to stimulate the vomiting center, though the relation between the abnormality and nausea cannot be established.

*Gibbs & Gibbs *reported in 1967 that in cases with paroxysmal slow activities, the occurrence rate of nausea or vomiting is higher than that of a normal subject group [39]. Other than this one, we could not find any other references indicating the direct relationship between EEG abnormality and nausea or abdominal distress. In the gynecology field, it was reported that in pregnant women with hyperemesis gravidarum during the first trimester, the frequency of abnormal EEG findings is significantly higher compared to that in pregnant women with no nausea and vomiting symptoms during pregnancy. These findings were also nonspecific [40]. The EEG abnormality could be evidence that some abnormal changes have occurred in the brain. Subjects with EEG abnormality might be sensitive to nausea.

2. Derealization (feelings of unreality) or depersonalization (being detached from oneself)

*Stein et al*. proposed the relation of these psychosensory symptoms to EEG abnormalities. However they were not able to clarify significant differences in their study. We considered that significant results could be gotten due to the large number of cases in our study. *Edlund *reported that four of six patients with atypical panic attacks involving hostility, irritability, severe derealization, and social withdrawal had temporal EEG abnormalities that could not be clearly considered as epilepsy [41].

3. Paresthesias (numbness or tingling sensations)

We could not find any references indicating a direct relationship between EEG abnormality and this symptom. However *Ietsugu et al*. reported that "Paresthesia," could be good indicator of severe panic attacks [42]. According to *Nishimura et al*, panic disorder patients with first-degree familial history (FH) are significantly younger at onset, show more symptoms, and have more frequent attacks with paresthesias and chills or hot flashes at first panic attack compared to patients without first degree FH [43]. We think that EEG abnormalities in panic disorder patients might have some relation to physiological predispositions that easily cause panic attacks. Accordingly, paresthesia might be a symptom that suggests EEG abnormality.

### Study limitations

Limitations in this study include the following:

1. There was a possibility that the EEG abnormality rate was high in the panic disorder patients in this study, although we can not determine this directly because of a lack of controls. Thus it will be necessary to reinvestigate comparing panic disorder patients and healthy people.

2. We grouped all EEG abnormality findings together and thus we can not refer to relationships between specific symptoms and specific EEG findings. Further studies with a larger number of subjects will be necessary to clarify our findings.

## Conclusion

In this study, we surveyed EEG abnormalities in panic disorder patients. 17 of the 70 cases examined had EEG abnormalities. Most abnormal findings were slow wave bursts and slow waves. These abnormalities were non-specific. Paroxysmal abnormality interpreted as epileptiform was found in only 2 of the 17 cases.

The following were extracted as factors related to EEG abnormalities in panic disorder: (i) nausea or abdominal distress, (ii) derealization (feelings of unreality) or depersonalization (being detached from oneself) and (iii) paresthesias (numbness or tingling sensations)

## Competing interests

The authors declare that they have no competing interests.

## Authors' contributions

MM carried out the revising the manuscript. MH carried out the statistical analysis and the revision of the manuscript. KN participated in the design of the study and the revision of the manuscript. KT carried out the revision of the manuscript. All authors read and approved the final manuscript
